# evalPM: a framework for evaluating machine learning models for particulate matter prediction

**DOI:** 10.1007/s10661-023-11996-y

**Published:** 2023-11-18

**Authors:** Lucas Woltmann, Jonas Deepe, Claudio Hartmann, Wolfgang Lehner

**Affiliations:** TU Dresden, Dresden Database Research Group, Dresden, Germany

**Keywords:** Particulate matter, Machine learning, Framework, Prediction

## Abstract

Air pollution through particulate matter (PM) is one of the largest threats to human health. To understand the causes of PM pollution and enact suitable countermeasures, reliable predictions of future PM concentrations are required. In the scientific literature, many methods exist for machine learning (ML)-based PM prediction, though their quality is difficult to compare because, among other things, they use different data sets and evaluate the resulting predictions differently. For a new data set, it is not apparent which of the existing prediction methods is best suited. In order to ease the assessment of said models, we present *evalPM*, a framework to easily create, evaluate, and compare different ML models for immission-based PM prediction. To achieve this, the framework provides flexibility regarding data sets, input features, target variables, model types, hyperparameters, and model evaluation. It has a modular design consisting of several components, each providing at least one required flexibility. The individual capabilities of the framework are demonstrated using 16 different models from the related literature by means of temporal prediction of PM concentrations for four European data sets, showing the capabilities and advantages of the *evalPM* framework. In doing so, it is shown that the framework allows fast creation and evaluation of ML-based PM prediction models.

## Introduction

Air pollution is a relevant problem and the cause for various health issues. Increased particulate matter (PM) pollution can lead to respiratory diseases and caused at least 238,000 premature deaths within the European Union (EU) in 2020 alone (European Environment Agency (2022)). Therefore, the EU and the World Health Organization (WHO) have set limits for PM concentrations that should not be exceeded to protect health. There is a distinction of $\text {PM}_{10}$, which consists of particles with a diameter $\le$ 10 $\mu$m and $\text {PM}_{2.5}$, particles of $\le$ 2.5 $\mu$m (World Health Organization (2021)). For $\text {PM}_{10}$, WHO recommends a limit of 45 $\mu$g/m³ as a daily average and 15 $\mu$g/m³ as an annual average (World Health Organization (2021)). For $\text {PM}_{2.5}$, the WHO recommended limits are 15 $\mu$g/m³ daily average and 5 $\mu$g/m³ annual average (World Health Organization (2021)). Compliance with the respective limit values should be permanently monitored with the help of spatially distributed measuring stations. These stations are classified according to their surroundings as *traffic*, *industrial*, or *background* stations and according to their positions in *urban*, *suburban*, or *rural* environments (European Environment Agency (2023b)).

In addition to measuring the current PM concentration, it is important to predict the expected PM concentration for the next day, e.g., in order to be able to inform the population about the air quality in good time and, if necessary, to be able to take countermeasures at short notice if limits are predicted to be exceeded (European Environment Agency (2011)). In addition, modeling PM concentrations enables the coverage of spatial gaps between measuring stations as well as the evaluation of planned measures to reduce PM pollution (European Environment Agency (2011)). In this context, *machine learning* (ML), which is a subcategory of artificial intelligence (AI), is suitable for the implementation of the corresponding models in several respects. ML encompasses a versatile set of powerful prediction methods that self-learn the potentially complex relationships using training data such that they do not need to be specified manually. Furthermore, using ML models is thus possible without fully understanding the underlying PM processes, such that the resulting models can even provide new insights regarding PM sources and distribution processes (European Environment Agency (2011)). This represents a significant difference compared to simulation methods, where the respective generation, distribution, and transformation processes must be explicitly defined in advance.

There is already a wide variety of approaches for ML-based prediction of PM concentrations in the scientific literature (Chae et al., 2021; Chang et al., 2020; Dhakal et al., 2021; Enebish et al., 2021; Kang et al., 2019; Karimian et al., 2019; Klingner & Sähn, 2008; McKendry, 2002; Nicklaß, 2010; Pérez, 2012; Pérez et al., 2000; Raimondo et al., 2007; Stadlober, 2013; Xayasouk et al., 2020; Zhao et al., 2023), but their quality is difficult to assess and compare since they each differ in several aspects. For example, Pérez et al. (2000) use linear regression and a feedforward neural network (FNN) to predict $\text {PM}_{2.5}$ concentrations in Santiago (Chile) in hourly resolution and evaluate the resulting models with a relative error measure. Karimian et al. (2019) use a gradient boosting machine, an FNN, and a long short-term memory (LSTM) model to predict $\text {PM}_{2.5}$ concentrations in Tehran (Iran) 12, 24, and 48 h into the future and evaluate the resulting models with absolute error measures, as well as the coefficient of determination $\text {R}^2$. This complexity of each work leads to a lack of comparability. To address the problem of lacking comparability between different works, this paper presents, as its core contribution, *evalPM*, a framework that can be used to easily create, uniformly evaluate, and compare different ML models for PM prediction. By implementing models from different works with the framework, their prediction quality can thus be effectively compared for any given data set.

The remainder of the paper is structured in the following way. In Sect. 1.1, we describe the overall problems that occur when predicting PM with ML models. Additionally, we derive requirements for an ML model framework to overcome these limitations. Next, we extensively compare and categorize related work that predicts PM concentrations with ML models in Sect. 1.2. Section 2 shows the overall structure of our *evalPM* framework, its components, and how they help to fulfill the framework requirements. In Sect. 3, we evaluate our framework according to the requirements with four different data sets. Lastly, we summarize this work and the merits of the framework in Sect. 4.

**Fig. 1:**
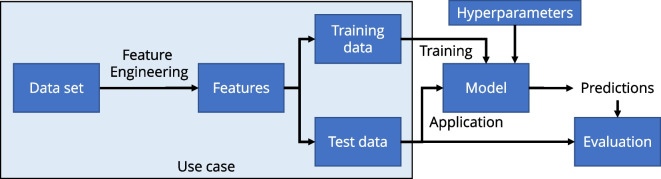
The process for ML-based PM prediction models detailing the connections between data set, use case, model, hyperparameters, training, and evaluation

### Problem statement

Existing works mainly describe ML methods for the prediction of PM concentrations using the same general process. An overview of this process can be found in Fig. 1. The general approach for ML-based PM prediction starts with the extraction of individual *features*, which are thought to have an influence on the PM concentration. Those features are used by a *model* to compute a *prediction of the target variable*, e.g., the average PM concentration of the next day. The internal parameters of the model that are used to conduct the calculations during the prediction process are learned using *training data*. During the *training process*, the model learns the connections between the features and the target variable. Therefore, it is important to have representative *training data* since connections that are not visible during training cannot be predicted with the model. The trained model is then *evaluated* using separate *test data* to check if the model generalizes to new and unknown data. There are different types of ML models that can be used for PM prediction. The structure and properties of such a model can each be controlled with model-specific *hyperparameters*. To compare the different data sets and their properties in related works, we define use cases. A *use case* within this work is defined as a combination of a data set with specific PM characteristics, the measurement variables available in the data set, the features calculated from the measurements, and the target variable to be modeled. A use case can also include the split of the data set into training and test data. For example, a use case would be the meteorology and PM measurements for a certain geographical region within a specific time range. It details components and the data flow for predicting PM concentrations in any use case.

Since the individual existing works differ in their objectives, their respective methods also differ in several aspects. On the one hand, the individual works differ in the data sets and the features calculated on them, as well as in the type of model used and the hyperparameters chosen for the model. On the other hand, they also differ in the modeled scenario or target variable, i.e., either $\text {PM}_{10}$ or $\text {PM}_{2.5}$ is modeled, with hourly or daily resolution and different prediction horizons, prediction of mean or maximum PM concentration or only binary prediction of the occurrence of a limit violation. In addition, the individual papers use different metrics to evaluate their models, such as the mean squared error (MSE) or the coefficient of determination $\text {R}^2$. However, the individual papers usually differ not only in one of these aspects but directly in several at once, so their results are not easily comparable. Some papers compare models with respect to one or more of these aspects, but they still usually cover only one or two use cases. Therefore, the results cannot be applied to other situations.

If a suitable prediction model is now sought for a new use case, it is not readily apparent which of the modeling approaches described in the literature is most suitable. On the one hand, the individual works use different data sets that are not necessarily representative of the new use case. Thus, the first requirement is that the described modeling approaches have to be tested on a new data set. Since not all measured variables used for the individual modeling approaches are necessarily available for the new use case, it must be possible to adapt the features calculated on the data set as a second requirement. At the same time, as a third requirement, it must also be possible to model different target variables since these can vary depending on the use case. On the other hand, the individual works also use different model types. Thus, as a fourth requirement, different model types must also be mapped with corresponding hyperparameters to evaluate different approaches. Finally, as a fifth requirement, the different models have to be evaluated consistently after their creation in order to select the best model. In this paper, we present and evaluate a framework which makes all these aspects possible. In summary, the framework enables the creation and comparison of PM prediction models given the following requirements:

1. Replacement of the used data set
2. Adjustment of the features calculated on the data set
3. Modeling of different target variables (namely $\text {PM}_{2.5}$ and $\text {PM}_{10}$)
4. Use of different ML model types with corresponding hyperparameters
5. Consistent evaluation of ML models

### Related work in PM prediction

Many works in the scientific literature have already dealt with the various aspects of PM prediction. To align these works with the prerequisites for our framework, we divide them into three fields: *feature selection* in Sect. 1.2.1, *machine learning (ML) models* in Sect. 1.2.2, and *hyperparameter optimization* in Sect. 1.2.3. This is followed by a summary in Sect. 1.2.5. The individual prediction models of selected papers are later compared in our evaluation using the framework presented in this work. They will show that we can adopt a variety of models and use cases with our framework.

#### Feature engineering

The feature engineering aspect is dedicated to investigating which input variables, i.e., features, are suitable for predicting PM concentrations.

Klingner and Sähn (2008) investigate which influence meteorological factors have on $\text {PM}_{10}$ concentrations. Their work focuses on the change in the current PM concentration and immission. For this, they use data from measuring stations in Saxony and Baden-Wuerttemberg, each covering a period of 6 years. They identify precipitation amount, wind speed, solar radiation, and the height of the atmospheric mixing layer as key influencing factors. In addition, they find that the $\text {PM}_{10}$ concentrations are more strongly influenced by solar radiation than by traffic volume. Based on these findings, the authors build a feed-forward neural network (FNN) model to predict the average $\text {PM}_{10}$ concentration of the following day based on meteorological features, day type (weekday, Saturday, Sunday, or holiday), and the $\text {PM}_{10}$ concentration of the previous day. However, since long-range meteorological measurement series usually do not include mixing layer heights, this feature is modelled by different temperature gradients in their model (Klingner and Sähn (2008)).

This great importance of meteorological factors for PM concentration is also supported by other work, both concerning $\text {PM}_{10}$ and $\text {PM}_{2.5}$. Stadlober (2013) identifies the same major factors as Klingner and Sähn (2008) for the $\text {PM}_{10}$ prediction in Graz (Austria) one day into the future using a linear regression (LR) model. In addition, as a further feature, the temperature difference to a higher altitude location is used as an indicator for temperature inversions. However, a nearby monitoring station with a significant elevation difference will rarely be available outside of mountains. Pérez et al. (2000) build FNN and LR models for hourly $\text {PM}_{2.5}$ predictions in Santiago (Chile) and detail that the addition of external meteorological features, like wind speed and humidity, improves the prediction quality of the models. Kang et al. (2019) use an LSTM model to predict $\text {PM}_{10}$ concentrations in Seoul (South Korea) up to 12 h into the future. Since they do not achieve satisfactory model quality with various air pollutant concentrations as features, they conclude that adding meteorological features would be beneficial.

Raimondo et al. (2007) use a feature elimination algorithm to automatically identify the optimal set of features for daily $\text {PM}_{10}$ predictions with FNN and support vector machines (SVM), respectively. They conduct the study on data from only one monitoring station in Gothenburg (Sweden) spanning a 2-year period. In addition to meteorological factors and previous $\text {PM}_{10}$ concentrations, they also identify ozone values as a relevant influencing variable. The importance of ozone values is also discussed by Chang et al. (2020) as a relevant feature for the hourly prediction of $\text {PM}_{2.5}$ concentrations in northern Taiwan. However, their prediction model exclusively covers elevated particulate matter concentrations to model limit violations.

Nicklaß (2010) uses a genetic algorithm to identify the most favorable feature selection for the prediction of the average $\text {PM}_{10}$ concentration on the following day for four measuring stations in Baden-Württemberg. As the most important features for the $\text {PM}_{10}$ prediction, it again identifies precipitation, wind speed, temperature, and the average $\text {PM}_{10}$ concentration of the previous day. However, the specific feature set differs by station and model type, so no general recommendation is given for features to use. Nicklaß also performs a sensitivity analysis regarding the uncertainty of weather predictions and finds that the quality of weather predictions is the most important factor in successful $\text {PM}_{10}$ predictions. McKendry (2002) also uses a genetic algorithm for feature selection in FNN and LR models for the daily prediction of the average and maximum $\text {PM}_{10}$ or $\text {PM}_{2.5}$ concentration in Chilliwack (Canada), arriving at results similar to those of Nicklaß (2010). However, McKendry’s resulting models are unable to adequately predict extreme PM concentrations.

#### Machine learning models

Since many different model types can be used to predict PM, some works investigate which model type is best suited for a specific application.

Nicklaß (2010) performs a comparison of several model types for daily $\text {PM}_{10}$ prediction in Baden-Württemberg. FNN achieves the best results, followed by SVM. Linear regression (LR) and a nearest neighbor approach (kNN) are less precise. However, it is emphasized that the kNN approach can also be used as a backup if not all regular features are available due to missing measured values. The superiority of an FNN as a nonlinear method over linear regression is also highlighted by Pérez et al. (2000) for hourly $\text {PM}_{2.5}$ prediction.

Pérez (2012) also builds a joint model for eight monitoring stations in Santiago (Chile) with 6 years of data to predict the maximum 24-h moving average $\text {PM}_{10}$ concentration on the following day. Here, an FNN with only one hidden layer is used to predict the numerical $\text {PM}_{10}$ concentration and clustering to predict the air pollution level. By combining the two models, Perez achieves greater accuracy in predicting high $\text {PM}_{10}$ concentrations than with the FNN alone. However, because this is a joint model for multiple stations, the result is not directly applicable to individual station models.

Enebish et al. (2021) compare six different model types for daily prediction of $\text {PM}_{2.5}$ concentrations in Ulaanbaatar (Mongolia). They use $\text {PM}_{2.5}$ data from nine stations, meteorological data from eight stations, each from 2010 to 2018, population data, and land use variables. However, from the $\text {PM}_{2.5}$ data, they only use the average concentration of the entire period under consideration as well as the warm season (April to September) and the cold season (October to March) as features, but not the respective concentration of the previous day. They compare the model types random forest (RF), gradient boosting machine (GBM), SVM, multivariate adaptive regression splines (MARS), generalized linear model with elastic net penalties (GLMNET), and generalized additive model (GAM) and create one variant each for the cold season, for the warm season, and for the entire period under consideration. The decision tree ensemble models (RF and GBM), which cover the entire period, achieve the best prediction quality.

Karimian et al. (2019) compare several types of models for hourly prediction of $\text {PM}_{2.5}$ concentrations at nine monitoring stations in Tehran (Iran). They use $\text {PM}_{2.5}$ data and meteorological data from a 4-year period and use the current $\text {PM}_{2.5}$ concentration, the current meteorological values, and the hourly meteorological values up to the prediction time (12, 24, or 48 h later) as inputs to the respective model. Here, a combination of FNN and long short-term memory (LSTM) achieves the best prediction quality, followed by a GBM and a classical FNN. Xayasouk et al. (2020) confirm the preeminence of LSTM models over FNN models on a 4-year data set of 25 stations in Seoul (South Korea) in hourly predictions of $\text {PM}_{10}$ and $\text {PM}_{2.5}$ predictions up to 10 days into the future.

Dhakal et al. (2021) compare LSTM models and a seasonal auto-regressive integrated moving average (SARIMA) model for daily prediction of $\text {PM}_{2.5}$ concentrations in the Kathmandu Valley (Nepal). They use data from seven stations whose daily readings are averaged into a single virtual station. The data are from January 2019 to May 2020, with only the last 31 days reserved for model testing. Dhakal et al. examine LSTM models with different feature combinations and prediction horizons, always using the past 20 days of feature data as input. The best prediction quality among the LSTM models is achieved by a model that predicts the $\text {PM}_{2.5}$ concentration on the immediate next day, using as features the concentration of $\text {PM}_{2.5}$ and the amount of dew from the previous days, respectively. This LSTM model achieves better prediction quality than the SARIMA model, which is modeled using past $\text {PM}_{2.5}$ values and does not use any other features in the prediction.

#### Hyperparameter optimization

Most model types have various hyperparameters that determine the model’s structure or learning behavior and thus influence the achievable model performance. In a neural network, for example, the hyperparameters include the number of intermediate layers, the activation function, and the learning rate. Other models have other hyperparameters directly affecting their prediction quality. However, in general, the optimal hyperparameters depend on the specific application of the respective model, so a separate hyperparameter optimization should be carried out for each application (Russell and Norvig (2020)).

To optimize the hyperparameters of kNN, FNN, and SVM, Nicklaß (2010) uses a grid search for categorical and integer parameters on the one hand and a genetic algorithm for real-valued parameters on the other hand. Raimondo et al. (2007); Xayasouk et al. (2020) also each use grid searches to optimize the hyperparameters of different model types. In Raimondo et al. (2007), it is also evident that selecting the optimal hyperparameters can more than double the probability of correctly predicting limit violations.

#### Model evaluation

Several mathematical metrics exist to evaluate the created prediction models. These describe the prediction error or agreement between the predicted values $\hat{y}$ and the actual values *y* within a data set with *n* data points. These are briefly described and defined in this section.

The *mean absolute error* (MAE) is a simple measure of prediction error and describes the absolute deviation of the predicted values from the actual values:

$$\begin{aligned} MAE(y,\hat{y}) = \frac{1}{n} \sum _{i=1}^n |y_i - \hat{y}_i |. \end{aligned}$$

The MAE considers all prediction errors equally, regardless of the error’s magnitude and the actual value’s magnitude.

The *symmetric mean absolute percentage error* (SMAPE) is an error measure that describes the percentage deviation of the predicted values from the actual values and is at most $200\%$:

$$\begin{aligned} SMAPE(y,\hat{y}) = \frac{200\%}{n} \sum _{i=1}^n \frac{|y_i - \hat{y}_i |}{|y_i |+ |\hat{y}_i |}. \end{aligned}$$

For the case $y_i = \hat{y}_i = 0$, the respective term of the sum is defined as 0. Since the SMAPE is a relative deviation, it is suitable for values with different ranges.

The *mean squared error* (MSE) is an error measure that describes the squared deviation of predicted values from actual values to give greater weight to larger deviations:

$$\begin{aligned} MSE(y,\hat{y}) = \frac{1}{n} \sum _{i=1}^n (y_i - \hat{y}_i)^2. \end{aligned}$$

Since the unit of MSE is not the same as the unit of the actual data, there is also the additional variant of *root mean squared error* (RMSE):

$$\begin{aligned} RMSE(y,\hat{y}) = \sqrt{MSE(y,\hat{y})}. \end{aligned}$$

The *coefficient of determination* $\text {R}^2$, unlike the previous metrics, is not a measure of prediction error but of prediction quality, i.e., a higher value (maximum 1) represents a better prediction.

$$\begin{aligned} R^2(y,\hat{y}) = 1 - \frac{\sum _{i=1}^n (y_i - \hat{y}_i)^2}{\sum _{i=1}^n (y_i - \bar{y})^2} \text { with } \bar{y} = \frac{1}{n} \sum _{i=1}^n y_i \end{aligned}$$

The coefficient of determination describes the proportion of variance in the actual values explained by the model.

The *relative directive error* (RDE) is defined by the European Environment Agency (2011) specifically for pollutant concentration predictions and always refers to the corresponding applicable limit value *G*.

$$\begin{aligned} RDE(y, \hat{y}, G) = \frac{|y_G - \hat{y}_G |}{G}. \end{aligned}$$

The value $y_G$ denotes the measured value closest to the limit, and $\hat{y}_G$ denotes the same-rank predicted value. If $y_G$ is the *k*-largest measured value, $\hat{y}_G$ is the *k*-largest predicted value. Thus, the RDE does not describe the prediction errors in the entire data set but only a slice related to the limit value.

**Table 1:** Comparison and classification of related work in the four most important categories according to an ML process

	Features	Models	Hyperparameters	Evaluation
Dhakal et al. (2021)	Meteorology,	LSTM, SARIMA	-	MAE, RMSE
	historical			
Enebish et al. (2021)	Meteorology,	GAM, GBM, GLMNET,	-	RMSE, $\text {R}^2$
	historical,	MARS, RF, SVM		
	population data,			
	land use			
Kang et al. (2019)	Other gases	LSTM	-	RMSE
Karimian et al. (2019)	Meteorology,	GBM, FNN, LSTM	-	MAE, RMSE,
	historical			$\text {R}^2$
Klingner and Sähn (2008)	Meteorology,	FNN	-	Visual
	historical,			
	type of day			
McKendry (2002)	Automatic	FNN, LR	-	RMSE, $\rho$
Nicklaß (2010)	Meteorology,	FNN, kNN, LR, SVM	Grid search,	MAE, RMSE
	historical		Genetic algo.	
Pérez et al. (2000)	Meteorology,	Clustering, FNN, LR	-	MAPE
	historical			
Raimondo et al. (2007)	Meteorology,	FNN, SVM	Grid search	Accuracy
	historical,			
	other gases			
Stadlober (2013)	Meteorology,	LR	-	Custom
	historical			
Xayasouk et al. (2020)	Historical	FNN, LSTM	Grid search	RMSE

All of these metrics can be calculated for both the training and test data sets. The evaluation of the test data set serves as an indicator of whether the model also generalizes to new data that was not used for training. Many works differ in their applied metric, making them difficult or even impossible to compare because the properties of the different metrics do not intersect. For example, many works use the RMSE as an evaluation metric (Dhakal et al., 2021; Enebish et al., 2021; Kang et al., 2019; Karimian et al., 2019; McKendry, 2002; Nicklaß, 2010; Xayasouk et al., 2020). However, many other works use different evaluation metrics that cannot be compared to the RMSE (Klingner & Sähn, 2008; Pérez et al., 2000; Pérez, 2012; Stadlober, 2013). The different metrics in use are listed in Table 1. This makes it challenging to find the best model for a data set or use case because different models can be best for different metrics. Additionally, it is not trivial to find the most important metric for a use case making the model selection even harder.

In contrast to these mathematical metrics, there are also domain-specific metrics. The modeling quality indicators (MQI) are a common metric as defined and used by the Forum for Air Quality Modeling (FAIRMODE), a joint initiative of the European Environment Agency and the European Commission Joint Research Centre.^1^ MQI were mainly developed for chemical transport models and their evaluation compared to measurements (Thunis et al. (2021)). They include the uncertainty of environmental measurements, allowing the models more freedom in their predictions. However, these indicators are seldom used for ML-based approaches because of their more complex definition compared to the mathematical metrics. Nonetheless, MQI are a well-established model evaluation method in the environmental research community.

#### Summary

In this section, some works have been presented that have dealt with the prediction of PM concentrations. All related works and their properties are compared in Table 1. In the feature engineering, meteorological factors have been found to be primarily relevant, in addition to previous PM concentrations. When comparing different types of models, neural networks have been the most popular, with FNNs being well complemented by LSTMs, especially for hourly predictions. In addition to neural networks, decision tree ensembles also provide good prediction performance. However, comparing different model types in the individual papers is always done for one specific use case only. Therefore, the results are not necessarily transferable to other use cases, as it was already addressed in the problem statement in Sect. 1.1. In addition, models are usually compared only based on individual metrics, which also differ from work to work. Optimization of model-specific hyperparameters is an important aspect of achieving the best possible prediction performance and can be performed by grid search or a genetic algorithm. However, the optimal hyperparameters depend on the particular use case, so hyperparameter optimization must be performed individually for each use case.

Besides the temporal aspect of PM prediction, the spatial distribution of particulate matter within a region, a country, or a continent can also be modeled, but this modeling of the spatial aspect is not the subject of this work.

## Materials and methods

The goal of the evalPM framework is to enable the creation, evaluation, and comparison of different variants of ML-based PM prediction models. The requirements for the framework and its structure are derived based on the problem statement in Sect. 1.1 and the related work in Sect. 1.2.

Since the model comparison should be performed on different use cases with data sets representative of the desired application, the framework must offer the possibility to exchange the underlying data set easily. The individual data sets can differ both in terms of the measurement variables they contain and their temporal scope. In order to be able to map different use cases, it must also be possible to calculate the desired target variable from the data set flexibly, e.g., as the mean or maximum value of the hourly $\text {PM}_{2.5}$ or $\text {PM}_{10}$ concentrations in different time granularity. Since different forecast models potentially use different features, the framework must allow flexibility in terms of the target variable as well as flexible, customizable feature calculation on the data set.

Orthogonally, the analyses may also differ in the type of ML model. Thus, the framework must support as many model types as possible or be able to be augmented with additional model types as needed. Since adjusting model type-specific hyperparameters is an integral part of model optimization and may differ between individual models, the framework must also support the specification of these hyperparameters for the different model types. The framework should also provide a way to optimize the hyperparameters of a model for a given use case in order to avoid the need for an additional tool for hyperparameter optimization, which should be performed separately for each use case.

For the evaluation of the created prediction models, some metrics exist that describe either the prediction error or the prediction quality. Since different metrics are used in the scientific literature, the framework should support as many of them as possible to establish points of comparison between the evaluated model and the different existing works. However, in addition to the comparison with individual metrics, a statistical test is also necessary to make robust statements about the significance of differences in model qualities. In addition, the framework should provide visualization capabilities for the data set and the predictions.

**Fig. 2:**
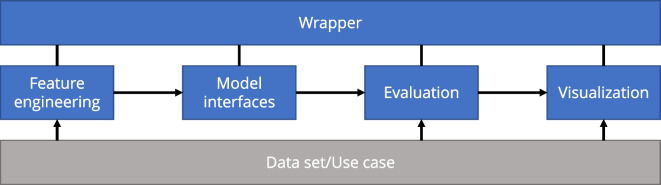
The structure of the *evalPM* framework detailing the connections between the components and the data flow

### Structure

The framework consists of several components, each fulfilling its own task and interacting with each other. The individual components are shown as an overview in Fig. 2 and are presented in more detail in the following.

**Data sets:** The data sets are not part of the actual framework but are necessary for the training and evaluation of ML models, so several data sets are provided alongside the framework. As described in the previous sections, the framework should provide the possibility to exchange the data set easily. To achieve this, it is only necessary to specify the location of the desired data set when using the evalPM framework. Thus, the framework can be adapted for new data sets without difficulties. Differences between individual data sets concerning the measured values and temporal scope are handled by the *feature engineering* component. The data sets are only expected to consist of one CSV file per measurement station with columns for the date, time, measurements, and target variables. However, the naming of the columns is freely selectable and can be specified during feature engineering.

**Feature engineering:** The *feature engineering* component is responsible for calculating features from the data set. On the one hand, to use data sets with different temporal scopes, the amount of training and test data can be adjusted by specifying the years to be used, respectively. On the other hand, in order to be able to use data sets with different measured variables and target variables, a reference to a file containing a description of the features to be calculated is also required. This feature description is done in JSON format and must contain the definition of the individual features and the designation of the date and time columns. Each individual feature is defined by a name, source attribute, aggregation details, and optionally a displacement.Aggregation is done by date by default, but for individual features, a different aggregation attribute can be specified using the optional group_by parameter within the aggregation block.

Also, the target variable is defined as one of the features and computed before it is separated from the actual features. This provides the same flexible calculation options for the target variable as for the features, allowing the modeling of different use cases on one data set.

For each measurement station in the data set, all defined features, including the target variable, are calculated individually for both the training and test data. Tuples are removed if they miss at least one feature. This is especially important for the beginning of the data sets, where features are calculated from previous values not contained in the data. Overall, this achieves both the flexibly customizable feature calculation on the data set and the modeling of different target variables. In addition to customizing or adding individual features, it is also possible to easily switch between different feature descriptions by referencing another corresponding JSON file.

**Model interfaces:** The *model interfaces* component acts as an interface between the framework and concrete model implementations of other libraries. This allows existing implementations of different ML models to be used and be available within the framework with a unified interface. Figure 2 gives an overview of the data flow from feature engineering to the model interfaces as well as the hyperparameters required and results produced in the process. Specifically, all model-specific *hyperparameters* are passed to the constructor of the respective model interface, and the resulting object provides two uniform methods for *training* the model and *prediction* with the model. The *training method* requires the appropriate feature data, including the target variable, to train the underlying model. The *prediction method* needs the corresponding feature data without the target variable to predict the target variable. The comparison between this prediction and the actual values of the target variable then takes place within the *evaluation* component. All methods include the possibility to measure their runtimes. This allows for benchmarking the different models according to efficiency. We can differentiate between training and prediction times.

Currently, the framework includes interfaces for the model types LR and GBM based on scikit-learn^2^ as well as for the model type FNN based on tensorflow.^3^ Adding further interfaces for other model types is possible in a straightforward way. Therefore, only the definition of the model interfaces must be adhered to in order to guarantee the usability within the evalPM framework. The framework also meets the requirement of supporting different model types with their corresponding hyperparameters, as formulated in Sects. 1.1 and 1.2.

**Evaluation:** The *evaluation* component is used to evaluate and compare model predictions. On the one hand, it offers possibilities to calculate different metrics, as introduced in Sect. 1.2.4. This requires the respective model predictions from the model interfaces component and the corresponding measured values or observations of the target variable from the data set. On the other hand, model comparison with the *almost stochastic order* test (Del Barrio et al. (2018); Dror et al. (2019)) is also implemented.

Based on the prediction quality of two models *A* and *B*, a cumulative distribution function (CDF) over the corresponding ratings is calculated for each model using any metric over several predictions. The principle of *stochastic order* expresses that a model is better than the other if its CDF is stochastically dominant, i.e., the CDF is lower than the other model’s CDF for each true value $y_i$ and prediction $\hat{y}_i$ under a certain metric. In practice, however, there is rarely a clear stochastic dominance as expressed by the mathematical metrics. In such cases, the *almost stochastic order* is superior to other metrics because it can be used to quantify the extent to which the stochastic order is violated, so one of the two models can still be declared better in the case of only a minor violation. For a classical metric, like the RMSE, this fine-granular differentiation is not possible. Specifically, using the CDFs of the two models *A* and *B*, a value $\epsilon _{\min }$ is computed that expresses an upper bound on the degree to which model *B* violates the stochastic dominance of model *A*. If $\epsilon _{\min } < \tau \le 0.5$, then model *A* is stochastically dominant over model *B* in more cases than vice versa, so model *A* can be said to be better. The smaller $\epsilon _{\min }$, the more certain is the superiority of model *A*. Here, $\tau = 0.2$ is recommended as an acceptance limit to achieve a balance between false positives and false negatives (Ulmer et al. (2022)), although the $\epsilon _{\min }$ result should always be included for the sake of transparency. Note, however, that $\epsilon _{\min }$ is not a *p* value, unlike other statistical tests, but the significance level $\alpha$ is already included in the calculation of $\epsilon _{\min }$.

To include the *almost stochastic order* in our framework, the implementation from the library deep-significance^4^ is used. The application of the test requires the predictions from two models to be compared, as well as the corresponding actual measured values of the target variable. Thus, the framework satisfies both the requirement to support different metrics and the requirement to compare models using a statistical test so that different models can be compared in a consistent manner.

**Visualization:** According to the requirements, the framework should provide functions for visual evaluation of results, which are based on the library matplotlib. The framework allows to display the predictions of one or more models and the corresponding actual measured values from the data set in a diagram. On the other hand, it is also possible to display evaluation metrics or results of the almost stochastic order test as a heat map.

**Wrappers:** The *wrapper function* component uses the model interface component and the evaluation component to implement both model training and prediction with the model and evaluation of the prediction with only one function call, thus simplifying the use of the framework. Within a function call, several model types and several hyperparameter variants per model type can be specified. The component also offers the possibility of hyperparameter optimization in the form of a grid search. Here, for each model or hyperparameter variant, the model interfaces component and the evaluation component are called once to train and evaluate a corresponding model. The grid search is also parallelized to shorten the runtime of the hyperparameter optimization.

### Summary

Based on the problem statement in Sect. 1.1 and the related work in Sect. 1.2 for a simple comparison of different ML-based PM prediction models, we presented the concrete implementation of the framework in this section. The framework consists of six components. The *data sets* are used for training and evaluation of models, have a flexible format, and can be easily exchanged. The *feature engineering* component computes features on the data set, flexibly defining the individual features in JSON format. The target variable, like $\text {PM}_{10}$ or $\text {PM}_{2.5}$, is also computed as one of the features to support different use cases. The *model interfaces* let the framework access existing implementations of different model types to train models and make predictions based on the feature data. The model types can be configured with hyperparameters, and interfaces to additional model types can be added. The *evaluation* component is used for uniform comparison of model predictions based on metrics or a statistical test. The *visualization* component enables the visual evaluation of results as a diagram or heat map. The *wrapper functions* build on other components and facilitate the use of the framework by allowing multiple model types and hyperparameter variants to be evaluated with a grid search.

The framework is publicly available as open source on GitHub^5^ and as a PyPI package^6^ to make it available for broad application in comparing ML-based PM prediction models. The repository on GitHub also includes a detailed documentation of the inner workings of evalPM and its components.

## Results and discussion

The main goal of the framework presented in this work is the ability to easily create, evaluate, and compare different variants of ML-based PM prediction models. In this section, we will demonstrate the fulfillment of the specific requirements for the framework, described in Sects. 1.1 and 1.2, by predicting various aspects of PM concentrations for different use cases. The first half of the evaluation presents the specific methodology before the second part presents the evaluation of the five framework requirements. Finally, Sect. 3.7 summarizes the merits of the framework according to the requirements.

### Setup

The detailed evaluation focuses on the results per region or data set, usually as an average over the individual station results of a region. Hyperparameter optimization experiments were performed on an AMD EPYC 7513 system with 16 cores and 128 GB RAM. All other experiments were performed on an Intel i5-8250U system with four cores and 8 GB RAM.

**Table 2:** Measurement stations for PM and meteorology and their classification (ARPA - Regione Lombardia (2023); Deutscher Wetterdienst (2023); European Environment Agency (2023a); The Norwegian Meteorological Institute (2023)). *bg* background, *tr* traffic

Region	Station name	Abbreviation	Station type	Weather station
Saxony	Chemnitz-Leipziger Str.	Ch-Leipz	Urban, tr	Chemnitz
	Collmberg	Collmberg	Rural, bg	Oschatz
	Dresden-Bergstr.	DD-Berg	Urban, tr	Dresden-Klotzsche
	Dresden-Nord	DD-Nord	Urban, tr	Dresden-Klotzsche
	Dresden-Winckelmannstr.	DD-WMS	Urban, bg	Dresden-Klotzsche
	Leipzig-Mitte	L-Mitte	Urban, tr	Leipzig/Halle
	Leipzig-West	L-West	Suburban, bg	Leipzig/Halle
Potsdam	Blankenfelde-Mahlow	Blankenfelde	Suburban, bg	Berlin Brandenburg
	Potsdam, Groß Glienicke	P-Glienicke	Suburban, bg	Potsdam
	Potsdam-Zentrum	P-Zentrum	Urban, bg	Potsdam
Lombardy	Casirate d’Adda	Casirate-dAdda	Rural, bg	Rivolta d’Adda Ist. Sp.
	Milano Pascal Città Studi	Milano-PCS	Urban, bg	Milano v.Juvara
	Milano Senato	Milano-Senato	Urban, tr	Milano v.Juvara
	Moggio	Moggio	Rural, bg	Cassina Valsassina
	Monza Machiavelli	Monza-Machiavelli	Urban, bg	Cinisello Balsamo
	Sesto San Giovanni	Sesto-San-Giovanni	Urban, tr	Cinisello Balsamo
Norway	Klosterhaugen	Klosterhaugen	Urban, bg	Skredderdalen
	Knarrdalstranda	Knarrdalstranda	Suburban, bg	Porsgrunn (Ås)
	Nedre Langgate	Nedre-Langgate	Rural, tr	Melsom
	Nygaardsgata	Nygaardsgata	Suburban, bg	Øsaker
	Våland	Valand	Suburban, bg	Sola

#### Data sets

For the creation and evaluation of PM prediction models, several data sets for different European regions were compiled within the framework. For reproducibility of the results, these data sets are published together with the framework. The framework can be used with different worldwide data sets. The focus on Europe stems from the availability of all data sources, including meteorological simulations.

Four regions are used as use cases as presented in Table 2 with the respective station type. The data set *Saxony* includes seven monitoring stations in the state of Saxony from 2010 to 2021. The data set *Potsdam* includes three monitoring stations in and around the city of Potsdam from 2016 to 2020. The data set *Lombardy* includes six measuring stations in the northern Italian region of the same name from 2017 to 2021 and serves as an example of a region with exceptionally high PM pollution. The data set *Norway* includes five measuring stations along the southern coast of Norway from 2017 to 2021 and differs from the other data sets due to its coastal location and more considerable distances between the measuring stations.

The individual data sets consist, in addition to the actual PM measurement values, of the meteorological measured variables air temperature, wind speed, precipitation amount, and an indicator for the type of day (working day, Saturday, Sunday, or holiday). Only the Saxony data set includes additional meteorological measured variables and air pollutants to provide corresponding features for comparison with other modeling approaches. If the meteorological measured variables are not collected by the respective PM measuring station, they were obtained from a nearby meteorological station. Most of the time, this is the closest meteorological station providing all the required measured variables. The assignment of the PM measuring stations to the respective weather stations used is listed in Table 2.

In the Saxony data set, almost all data come from *Saxony State Office for Environment, Agriculture and Geology* (LfULG) (für Umwelt, Landwirtschaft und Geologie (2023)), only the precipitation data come from *Deutsche Wetterdienst* (DWD) (Deutscher Wetterdienst (2023)). In the Brandenburg data set, the PM data come from *Luftgütemessnetz Brandenburg* (Ministerium für Landwirtschaft, Umwelt und Klimaschutz (MLUK) des Landes Brandenburg (2023)) and the meteorological data from DWD. In the Lombardia data set, both the PM data and the meteorological data come from the regional environmental protection agency *ARPA Lombardia* (ARPA Lombardia (2023a, 2023b)). In the Norwegian data set, PM data are from *European Environment Agency* (EEA) (European Environment Agency (2023c)), and meteorological data are from *Norwegian Meteorological Institute* (MET Norway) (The Norwegian Meteorological Institute (2023)). The underlying data were not cleaned to create the data sets, as the respective authorities have already validated them.

According to some works (Klingner and Sähn (2008); Nicklaß (2010)), in addition to temperature, wind speed, and precipitation, the height of the atmospheric mixing layer is also a significant influencing factor for PM concentration. Therefore, we calculate it as an additional meteorological parameter using the *ERA5 data set* in order to be able to use it as a feature in the models. The ERA5 data set is produced by the *European Centre for Medium-Range Weather Forecasts* (ECMWF) using an analysis of global weather and climate. It includes various meteorological parameters as raster data with an hourly resolution, available in the *Copernicus Climate Change Service* (Copernicus Climate Change Service, Climate Data Store (2023); Hersbach et al. (2023)). Atmospheric mixing layer heights were calculated from this data using the algorithm described by ECMWF (European Centre for Medium-Range Weather Forecasts (2017)). However, since the ERA5 data are raster data with $0.25^\circ$ horizontal resolution, the calculated values are not necessarily representative of the exact location of the respective measurement station. In addition, the calculated mixing layer heights can only be an approximation of the actual values for the respective grid cell since they are calculated using data from a few discrete height layers.

In addition to the various measured values, a data set with PM predictions based on numerical simulations is also available (European Centre for Medium-Range Weather Forecasts (n.d.)) for comparing the ML models to a traditional approach. This consists of hourly predictions from the *European Air Quality Forecast* in the *Copernicus Atmosphere Monitoring Service*, averaged to daily values (Copernicus Atmosphere Monitoring Service (CAMS), Atmosphere Data Store (2023)). The purpose of this data set is to compare the long-range PM prediction for all of Europe with the local PM prediction at a single monitoring station. Specifically, the data set includes predictions from the models *EURAD-IM* (German) and *EMEP* (Norwegian), as well as the median of all participating models (*Copernicus ensemble*) (FRANCE et al. (2022); für Energie-und Klimaforschung (IEK) (2020); Norwegian Meteorological Institute (MET Norway) (2020)). The Italian model *MINNI* has only been part of the ensemble since June 2022 and was thus not available within the scope of this work (European Centre for Medium-Range Weather Forecasts (n.d.)). Since the European Air Quality Forecast is raster data with $0.1^\circ$ horizontal resolution, it is not necessarily representative of the exact location of the respective measuring station, similar to the ERA5 data.

#### Implemented models

To evaluate our framework, different modeling approaches are evaluated on the individual data sets to demonstrate the framework’s capabilities with respect to model comparison. The respective modeling approaches are based on individual related works presented in Sect. 1.2 but had to be partially adapted to the four data sets. In addition to the modeling approaches of related work, hyperparameter optimization is also performed per data set for the model types *linear regression* (LR), *gradient boosting machine* (GBM), and *feedforward neural network* (FNN). The best model variant after hyperparameter optimization in each case is also included in the model comparison, as well as the PM predictions of three models of the European Air Quality Forecast (Copernicus Atmosphere Monitoring Service (CAMS), Atmosphere Data Store (2023)) based on numerical simulations. The remainder of this section lists the models of related work used for evaluation and the respective adjustments to the specific modeling approach.

The FNN model of Klingner and Sähn (2008) is used to predict both $\text {PM}_{10}$ concentrations and $\text {PM}_{2.5}$ concentrations, although initially only designed for $\text {PM}_{10}$ concentrations. However, because $\text {PM}_{2.5}$ is a subset of $\text {PM}_{10}$, it can be assumed that a $\text {PM}_{10}$ modeling approach is also applicable to $\text {PM}_{2.5}$ prediction without much loss of quality.

From Nicklaß (2010), all four model types are used: LR, *k-nearest neighbors* (kNN), *support vector machine* (SVM), and FNN. Again, these models are used for the prediction of $\text {PM}_{10}$ concentrations, but in the context of our framework, also for the prediction of $\text {PM}_{2.5}$ concentrations. The following selection is made since Nicklaß partially optimizes the selected features and hyperparameters depending on the station. Only features used by Nicklaß at a majority of stations are used per model type, and the hyperparameter *k* of the kNN model is set to $k=10$ as per the author’s recommendation.

From McKendry (2002), both the LR and the FNN models are used to predict the average $\text {PM}_{2.5}$ concentration of the following day. For the FNN model, an intermediate layer with ten neurons and sigmoid activation is assumed because McKendry does not explicitly specify hyperparameters. In addition, the features of the maximum and standard deviation of $\text {PM}_{2.5}$ concentrations for a day can only be used for data sets that contain $\text {PM}_{2.5}$ concentrations with hourly rather than daily resolution, so these features are missing for the Saxony and Lombardy data sets.

From Enebish et al. (2021), the RF, GBM, and SVM models are used, and spatial features are omitted because only temporal PM predictions are considered.

From Dhakal et al. (2021), both the SARIMA model and the best LSTM model are used, with the dew feature replaced by the precipitation sum, since no dew readings were available in sufficient volume. In addition, as a simple time series forecast, the SARIMA model is updated daily with the current measured values, as Dhakal et al. do not specify any required information about this. In addition, the scaling in the lambda layer of the LSTM model is dynamically adjusted to the maximum PM concentration in the training data for each monitoring station since Dhakal et al. build their LSTM model statically for a single (virtually aggregated) monitoring station.

From Pérez et al. (2000), the LR model and the basic FNN model are used to predict $\text {PM}_{2.5}$ concentrations on an hourly basis. A separate model is trained for each hour of the day, and the daily predictions from all 24 models are averaged to predict the daily average. Since this method is only applicable to data sets containing $\text {PM}_{2.5}$ concentrations with hourly rather than daily resolution, modified models are used for data sets with daily resolution of $\text {PM}_{2.5}$ concentration, i.e., Saxony and Lombardy. Instead of predicting an hourly value of $\text {PM}_{2.5}$ concentration using the 24 hourly values of the previous day, the daily value of $\text {PM}_{2.5}$ concentration is predicted using the daily values of the 24 previous days.

**Table 3:** Data set flexibility shown by applying one model (Klingner-FNN) to all four data sets and comparing its quality according to all five metrics. For better comparison, the average $\text {PM}_{2.5}$ and its standard deviation are given for each data set

Data set	$\varnothing$ $\text {PM}_{2.5}$ $[\frac{\mu g}{m^3}]$	MAE $[\frac{\mu g}{m^3}]$	RMSE $[\frac{\mu g}{m^3}]$	SMAPE (%)	R²	RDE (%)
Saxony	9.67± 6.38	3.01	4.34	32.44	0.52	8.21
Potsdam	9.09±6.10	2.74	3.86	35.85	0.60	9.95
Lombardy	18.53±13.17	4.70	6.64	34.18	0.73	9.43
Norway	7.88±6.46	2.95	4.39	39.66	0.35	22.90

Not all originally used features are available for each modeling approach since the four data sets do not contain all corresponding measured variables. However, as an example, the *Saxony* data set was supplemented with all required metrics so that a comparison of the models with the original features versus the respective models with restricted features can be made on this data set. Furthermore, unless otherwise described, for all models, the data of one calendar year are used as training data and the data of the following calendar year are used as test data, regardless of how many training and test data were originally used in the respective modeling approach. Specifically, for the Saxony, Lombardy, and Norway data sets, data from 2020 is used for training and from 2021 for testing the models. For the Potsdam data set, on the other hand, data from 2019 is used for training and from 2020 for testing the models, as this data set does not contain any data from 2021.

#### Parameters of statistical tests

Model comparisons are performed using the *almost stochastic order* test. Following the recommendation of Ulmer et al. (2022), $\tau =0.2$ is used as a threshold for the significance of the superiority of one model over another. Moreover, the error level $\alpha =0.05$ is already included in the $\epsilon _{\min }$ calculation.

The individual squared errors of all stations in a region are used as evaluations of the models used to calculate the cumulative distribution functions for model comparison. The prediction errors from each individual prediction across all stations in a region are used to provide as many values as possible per model for the approximation of the cumulative distribution functions. The individual errors are squared to give greater weight to larger errors.

**Fig. 3:**
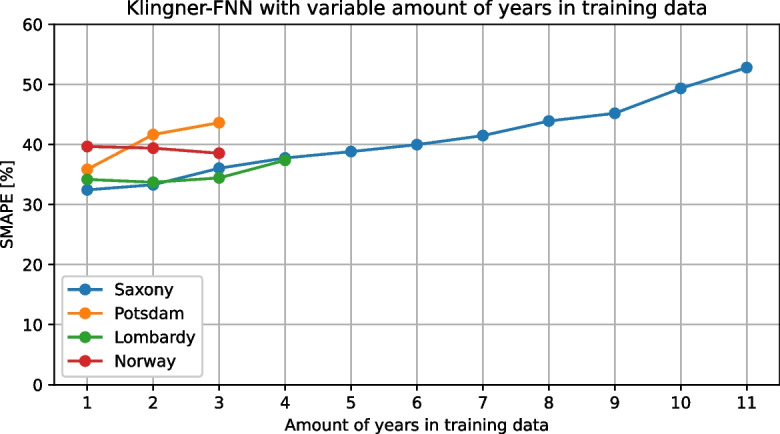
The influence of the number of years in training data on the model quality (Klingner-FNN) for all four data sets

### Data set flexibility

The first requirement for the framework is the ability to easily exchange the data sets to model representative use cases. To demonstrate this, we use one model, the FNN model of Klingner and Sähn (2008) (Klingner-FNN), on each of the four data sets to predict the daily $\text {PM}_{2.5}$ concentration. The results of this experiment are presented in Table 3 with the different metrics described in Sect. 1.2.4. To classify the values of MAE and RMSE, the table also includes the mean and standard deviation of the $\text {PM}_{2.5}$ concentrations of each data set, as these two metrics also depend on the magnitude of the target variable and are thus limited in their comparability between different data sets. The Klingner-FNN produces good values for all metrics over all data sets. It is able to model and predict all necessary data points in the test sets. This reinforces our goal that every model implemented in the framework is transferable to a variety of different data sets. Besides that, Table 3 shows that the mathematical metrics do not exhibit regional bias. All four data sets show different mean values and standard deviations for their measurements, but the ranges of the metrics remain similar. The only exception is the RDE for the Norwegian data set, but we argue that this results from the low $\text {PM}_{2.5}$ concentrations for these stations. Therefore, little data around the limit value is available, skewing the RDE.

In addition, to be able to use data sets with different temporal scales, it is possible to specify in the framework which specific years from the data set should be used for training or testing the model. To demonstrate this functionality, the Klingner-FNN was again used on all four data sets to predict the daily $\text {PM}_{2.5}$ concentration, gradually increasing the number of years used for training as much as possible. The test year for each data set remained constant, and the appropriate number of years immediately preceding the test year was used to train the model. The results of this experiment are shown in Fig. 3 using the SMAPE. It can be seen that the model quality for the Saxony and Potsdam data sets decreases (i.e., the model error increases) as the number of training years increases, while for Lombardy, it increases at least from 1 to 2 years, and for Norway, it also increases again from 2 to 3 years. With an increasing amount of training data, the respective model can theoretically learn from more situations. However, the simple model structure might not process this additional information advantageously. Therefore, the hyperparameters of the model would have to be adjusted accordingly. On the other hand, data from far back could potentially describe relationships that are no longer applicable, like changes in the sensor’s local environment, e.g., building development or traffic routing. These changes can have a negative impact on the prediction quality. In conclusion, it is easily possible to represent different use cases with data sets and features with the *evalPM* framework. The framework works data-independently and can be used for any PM measurement data sets.

### Feature flexibility

**Fig. 4:**
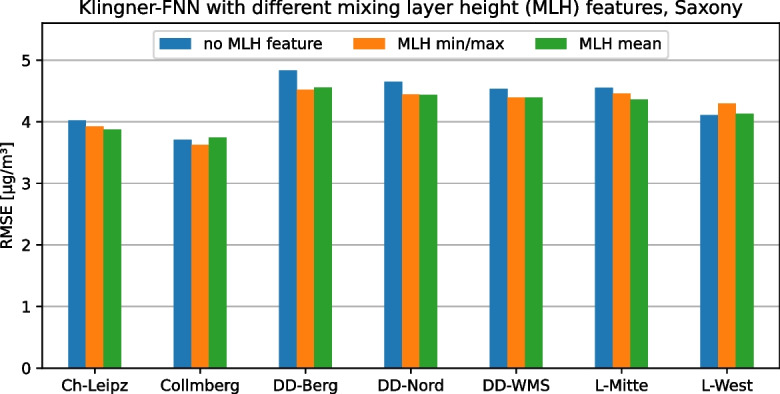
The inclusion and replacement of features and their effect on the model quality (Klingner-FNN) for all stations in the Saxony data sets

The second requirement for the framework is the possibility to flexibly adapt or extend the features calculated on the data set in order to be able to implement different modeling approaches with their respective individual features or to be able to adapt them to the measured quantities available in the respective use case. To demonstrate this functionality, different features of the mixing layer height (MLH) were added to the Klingner-FNN as an example because according to Klingner and Sähn (2008), it is a major factor influencing PM concentration, but is not used in their prediction model. Specifically, the prediction of daily $\text {PM}_{2.5}$ concentrations on the Saxony data set was performed once with the original Klingner-FNN (without MLH), once with the daily mean MLH as an additional feature, and once with the daily minimum and maximum MLH as two additional features. The results of this experiment are shown in Fig. 4 using the RMSE for each station in the Saxony data set, respectively. Depending on the station, the MLH features have a different effect on the RMSE of the model but averaged across the seven stations, the model variant with the MLH mean feature the lowest RMSE (4.21 $\mu$g/m³), followed by the variant with MLH minimum and maximum (4.24 $\mu$g/m³). Thus, on average, the two variants with MLH features each represent a slight improvement over the original variant of Klingner et al. (4.34 $\mu$g/m³). Furthermore, a more concrete statement could be made using a statistical model comparison.

This experiment enforces our argument that feature engineering is important for PM prediction model selection and a necessary requirement for our framework. Therefore, our framework supports feature flexibility to allow conclusions about the influence of different features on the models and their quality. Additionally, feature flexibility helps to find the optimal feature set for a use case by testing different feature combinations and selecting the best one.

**Fig. 5:**
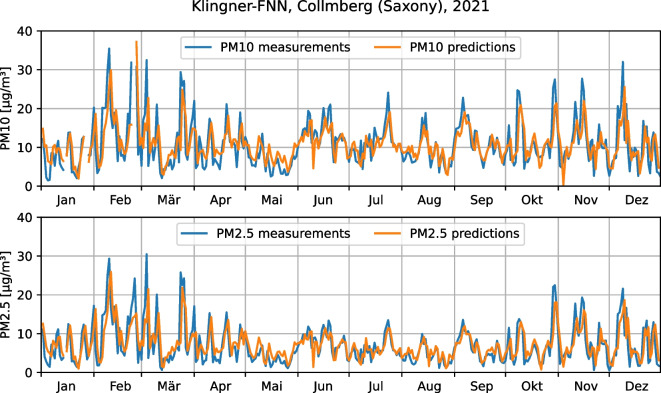
The evaluation of the capability to predict different target variables ($\text {PM}_{2.5}$ and $\text {PM}_{10}$) with one model (Klingner-FNN) for station Collmberg, Saxony

### Target variable flexibility

The third requirement for the framework is the ability to model different target variables to represent different use cases. To demonstrate this functionality, the Klingner-FNN was used to predict the daily concentration of $\text {PM}_{10}$ on the one hand and $\text {PM}_{2.5}$ on the other hand as the target variable on the Saxony data set. Therefore, a separate model was trained and tested per station and target variable. Since the $\text {PM}_{10}$ data in the Saxony data set is not completely available for the station *Chemnitz-Leipziger Str.*, this station was excluded from this experiment.

The results of this experiment are shown as an example for the station *Collmberg* in Fig. 5. It can be seen that the measured concentrations of $\text {PM}_{10}$ and $\text {PM}_{2.5}$ follow a very similar course, with $\text {PM}_{2.5}$ concentrations always being slightly lower than the respective $\text {PM}_{10}$ concentrations. This is because $\text {PM}_{2.5}$ is always a subset of $\text {PM}_{10}$. On the other hand, it can be seen that the models’ predictions also follow the course of the respective measurements well, only especially high values tend to be underestimated. This underestimation of high concentrations has also been described by Nicklaß (2010) for different model types and could be a result of the under-representation of high concentrations in the training data, as they only make up a small fraction, whereas medium concentrations are represented much more frequently.

**Fig. 6:**
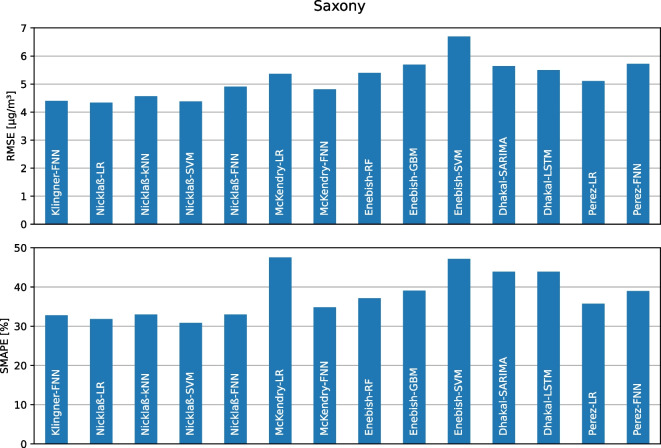
The average qualities (RSME and SMAPE) of different models on the Saxony data set

Based on the good visual agreement of the predictions with the respective measurements of both $\text {PM}_{10}$ and $\text {PM}_{2.5}$ and the similar error values of the models, it can be concluded that the Klingner-FNN, which was originally designed for $\text {PM}_{10}$ prediction only, is also suitable for $\text {PM}_{2.5}$ prediction. This is because $\text {PM}_{2.5}$ and $\text {PM}_{10}$ are both affected by very similar immission processes. Moreover, the magnitudes of $\text {PM}_{10}$ and $\text {PM}_{2.5}$ concentrations are close, at least for the Saxony data set. However, to ensure general implications, more experiements are necessary. Nevertheless, we can conclude that our framework is capable of predicting and modeling different target variables. With $\text {PM}_{2.5}$ and $\text {PM}_{10}$, we model two environmental measurements. However, our framework is not limited to these two variables. It can be used for any environmental variable that can be represented in a numerical value.

### Model flexibility including hyperparameters

The fourth requirement for the framework is the ability to use different model types with their respective hyperparameters and optimize these hyperparameters to determine an optimal prediction model for the particular use case. To demonstrate this functionality, the models described in Sect. 3.1.2 from related work were used to predict the daily $\text {PM}_{2.5}$ concentration on the Saxony data set. Because the additional meteorological metrics and air pollutants in the Saxony data set are not fully available for the *Chemnitz-Leipziger Str.* station, this station was excluded from this experiment for all models. The results of this experiment are shown in Fig. 6 using the average RMSE and SMAPE per model. Here, the RMSE varies by model from 4.34 $\mu$g/m³ for the LR model of Nicklaß (2010) (Nicklaß-LR) to 6.70 $\mu$g/m³ for the SVM model of Enebish et al. (2021) (Enebish-SVM). Even within a model type, model quality varies by different hyperparameters or features used, e.g., for FNN models in terms of RMSE from 4.40 $\mu$g/m³ (Klingner-FNN) to 4.81 $\mu$g/m³ and 4.90 $\mu$g/m³ (McKendry-FNN and Nicklaß-FNN) to 5.72 $\mu$g/m³ (Perez-FNN). The SMAPE varies between the individual models and within the respective model types to a similar extent as the RMSE. For the best possible model quality, the selection of suitable hyperparameters and features is, therefore, important in addition to the model type.

**Table 4:** Overview over different model hyperparameters and their optimization ranges

Model type	Hyperparameter	Ranges	Saxony	Potsdam	Lombardy	Norway
Optimized LR	Weights	{lin., unif., exp}	unif.	unif.	unif.	unif.
Optimized GBM	No. trees	[5, 100]	85	30	45	95
	Max. depth	[1, 7]	2	5	3	3
	Min. leaf tuples	[1, 100]	5	20	5	1
Optimized FNN	Width	[4, 16]	16	16	16	12
	Depth	[1, 4]	3	1	4	1
	Activation	{linear, ReLU}	ReLU	ReLU	ReLU	ReLU
	Dropout rate	{0%, 30%, 50%}	0%	0%	0%	0%
	Batch size	{16, 32}	16	32	32	16

For all four data sets, the best (optimized) hyperparameters for the three models are shown

Therefore, starting from the modeling approach of Klingner and Sähn (2008), a hyperparameters optimization was performed for the model types LR (optimized LR), GBM (optimized GBM), and FNN (optimized FNN). The parameter space for the corresponding grid search is shown per model type in Table 4. For the LR model type, there are no hyperparameters to be optimized. For each hyperparameter combination, the model is trained and then applied to the test data. The best combinations of hyperparameters, in terms of a minimum RMSE, for all data sets are listed for optimized GBM and FNN in Table 4. For the Saxony data set (excluding the station *Chemnitz-Leipziger Str.*), the optimized GBM model delivers the best model quality with an RMSE of 4.05 $\mu$g/m³, undercutting the best model of related work (Nicklaß-LR with 4.34 $\mu$g/m³). Additionally, the optimized FNN reaches an RMSE of 4.1 $\mu$g/m³, which is also better than the models from related work, but not the best compared to the optimized GBM. This result supports the high importance of hyperparameter optimization because it influences model quality. A sub-optimal hyperparameter selection for one model can make it worse than another, even though it would produce the best results for a use case with the right hyperparameters. This highlights the importance of model and hyperparameter flexibility in our framework. With this capability, the framework can find the best model for a use case. Additionally, it can automatically determine the best hyperparameter configuration for any model type, increasing prediction qualities even further.

**Fig. 7:**
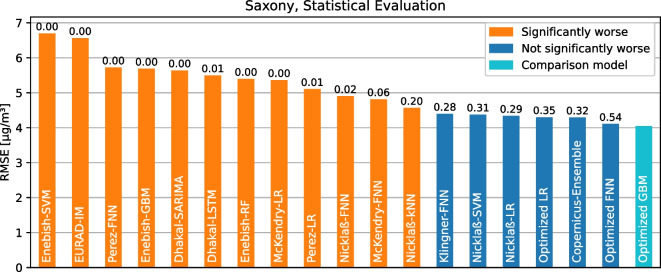
The statistical comparison if models are worse on the Saxony data set than the optimized GBM based on the RMSE and the almost stochastic order test. $\epsilon _{min}$ values are shown above the bars

**Fig. 8:**
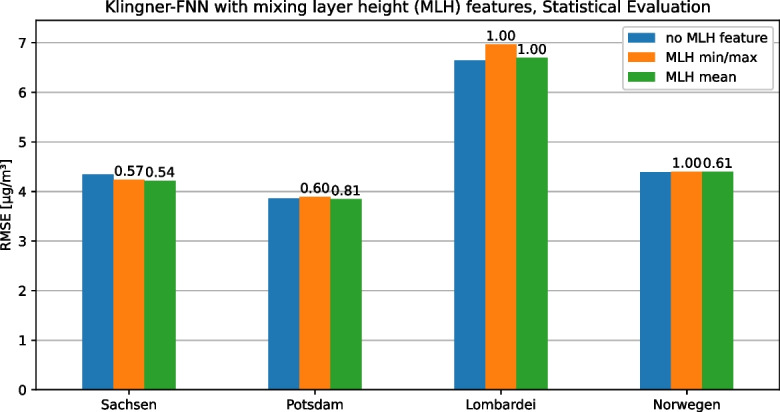
The statistical comparison of the effect of different mixing layer height (MLH) features on the Kligner-FNN with the Saxony data set based on the RMSE and the almost stochastic order test. $\epsilon _{min}$ values are shown above the bars

### Model evaluation

The fifth and final requirement for the framework is the ability to evaluate different models uniformly and compare their prediction quality. The evaluation using various metrics defined in Sect. 1.2.4 and graphical comparison of measurements and predictions has already been demonstrated in detail throughout this section, so we focus on model comparison using statistical testing. The *almost stochastic order* test is used for this purpose, which was explained in more detail in Sect. 2.1.

As a first experiment, all considered models are evaluated in more detail on the Saxony data set. Since the additional meteorological metrics and air pollutants in the Saxony data set by some models were not fully available for the station *Chemnitz-Leipziger Str.*, this station was again excluded from this experiment for all models. Specifically, the statistical test is used to check whether the model with minimum RMSE (the optimized GBM model) is significantly better than each of the other models. The results of this experiment are shown in Fig. 7, where the models are sorted by their average RMSE on the Saxony data set. According to the threshold $\tau =0.2$, the optimized GBM model is thereby significantly better than some models of related works, but not significantly better than the LR and SVM models of Nicklaß (2010), the FNN model of Klingner and Sähn (2008), the Copernicus ensemble (FRANCE et al. (2022)), or the other optimized models. Due to rounding to two decimal points, $\epsilon _{\min }=0.20$ is shown for the kNN model of Nicklaß (2010), but the value is actually $\epsilon _{\min }=0.1955$ and thus is genuinely smaller than $\tau$, as required for a significant result. It should be emphasized that the $\epsilon _{\min }$ values within a data set do not necessarily increase monotonically with decreasing RMSE. For the Norway data set, the FNN model of Pérez et al. (2000) with $\epsilon _{\min }=0.08$ is even significantly worse than the model with minimum RMSE (Perez-LR), although some models with larger average RMSE are not significantly worse, such as the Nicklaß FNN with $\epsilon _{\min }=0.24$. This again illustrates that evaluation by a simple metric such as RMSE is insufficient to compare models and that details are lost by averaging over multiple predictions. In this case, it is beneficial to use a statistical test, as implemented in our framework.

Additionally, the three different feature variants of the Klingner-FNN are evaluated on all four data sets as a second experiment. The three variants are those of Klingner and Sähn (2008) without mixing layer height (MLH) and with MLH mean or MLH minimum and maximum. In addition, the statistical test is used to check whether the variants with MLH features are significantly better than the variant without MLH features in each case since the RMSE on the Saxony data set points in this direction and since Klingner and Sähn (2008) identify MLH as a significant factor influencing PM concentration. The results of this experiment are shown in Fig. 8. In this, it can be seen that none of the $\epsilon _{\min }$ values fall below the threshold $\tau =0.2$, so for none of the four data sets, an MLH variant is significantly better than the original variant without MLH features.

**Fig. 9:**
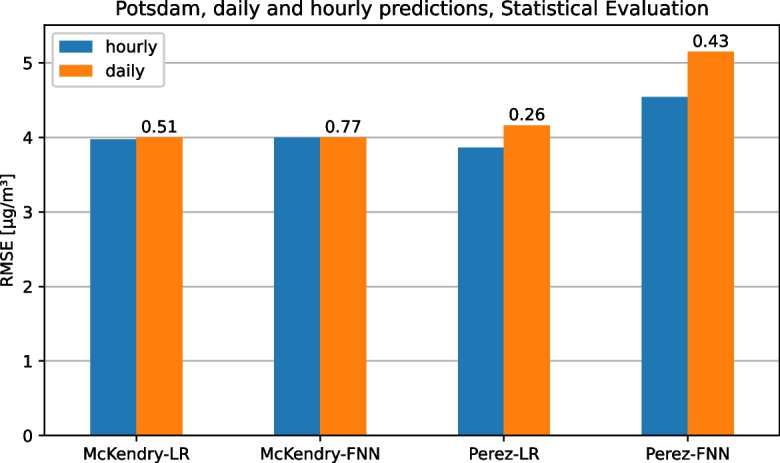
The statistical comparison of different models for daily and hourly predictions for the Potsdam data set based on the RMSE and the almost stochastic order test. $\epsilon _{min}$ values are shown above the bars

**Fig. 10:**
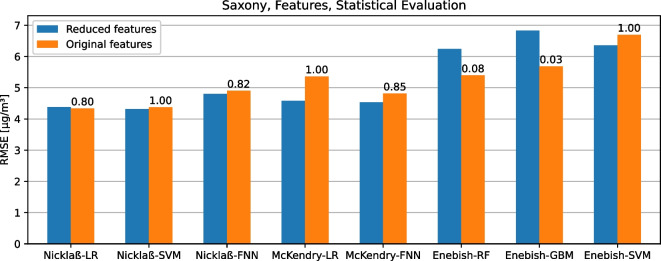
The statistical comparison of reduced feature sets for different models with the Saxony data set based on the RMSE and the almost stochastic order test. $\epsilon _{min}$ values are shown above the bars

Because the Saxony and Lombardy data sets contain $\text {PM}_{2.5}$ data only at daily resolution and not at hourly resolution, the models of McKendry (2002); Pérez et al. (2000) for these data sets had to be adapted accordingly. Therefore, as a third experiment, we evaluate what impact these adaptations have on the model quality by testing both the original and the adapted models. Furthermore, the statistical test checks whether the models with hourly $\text {PM}_{2.5}$ data are significantly better than the respective models with daily $\text {PM}_{2.5}$ data. The results for the Potsdam data set are shown in Fig. 9. It can be seen that the adjustments have almost no effect on the models of McKendry (2002), while the models of Pérez et al. (2000) have a sometimes significantly larger RMSE due to the adjustment to daily $\text {PM}_{2.5}$ data. However, using the $\epsilon _{\min }$ values, it is clear that the original models with hourly $\text {PM}_{2.5}$ data are not significantly better than the fitted models with daily $\text {PM}_{2.5}$ data for any of the combinations of model and data set. This again illustrates that when models are compared using a simple metric such as the RMSE, details are lost that can be used by a detailed statistical test to achieve more meaningful results.

In addition to the prediction of $\text {PM}_{2.5}$ data, some models also have to be adapted to the measured variables available in the data sets. For example, additional air pollutants and meteorological metrics were added to the Saxony data set in order to be able to evaluate the corresponding models on them using the original features described in the respective paper. As a fourth experiment, these models are evaluated with adapted or restricted features, as required by the other data sets, and with the original features on the Saxony data set. The station *Chemnitz-Leipziger Str.* could again not be included because not all relevant additional measured variables were available for this station. Therefore, this experiment also excludes it from the models with restricted features. In addition to the evaluation using the RMSE, the statistical test is also used to check whether the models with original features are significantly better than the respective models with restricted features. The results of this experiment are shown in Fig. 10. It can be seen that depending on the model, the adaptation or restriction of the features leads partly to an improvement and partly to a deterioration of the model quality. Only the models Enebish-RF and Enebish-GBM are significantly better with the original features than with restricted features, whereas the model McKendry-LR is even significantly better with restricted features than with the original features ($\epsilon _{\min }=0.004$).

### Summary

This section demonstrates the fulfillment of the requirements for the framework presented in this paper. The framework’s essential goal is to easily create, evaluate, and compare different variants of ML-based PM prediction models. The concrete requirements derived in Sects. 1.1 and 1.2 can be summarized as flexibility with respect to the data set, the features, the target variables, the model type, including its hyperparameters, and model evaluation. To demonstrate the relevant framework capabilities, the modeling approaches of some related work were implemented and applied on four different European data sets to predict $\text {PM}_{10}$ or $\text {PM}_{2.5}$ concentrations. In addition, hyperparameter optimization for different model types was performed for each data set, and a statistical test was used to compare the individual models. In particular, the importance of the statistical test with our framework for a meaningful model comparison became apparent in several experiments.

## Conclusion

In the scientific literature, many approaches for predicting PM concentrations using ML already exist, but it is difficult to compare their quality because they model different data sets, use different model types, and evaluate the resulting predictions differently. Therefore, this work presented the framework *evalPM*, which can be used to easily create, evaluate, and compare ML prediction models. In order to reproduce modeling approaches from the literature and create new models for different use cases, the following requirements are fulfilled by the framework. It is possible to replace the data sets to apply existing models to various use cases. Features can be customized to enhance existing models or create new models. The framework allows the modeling of different target variables to be adaptable to different environmental measurements. Due to the universality of the framework and ML models, this is not limited to air pollutants. The users can use different model types with appropriate hyperparameters to fit their use case. Lastly, we introduced a uniform evaluation of models, including a statistical comparison.

The aforementioned capabilities of the framework were demonstrated using 16 different models from the related literature by means of temporal prediction of PM concentrations for four European data sets. Both $\text {PM}_{10}$ and $\text {PM}_{2.5}$ concentrations were modeled, different feature variants were used, and hourly and daily PM predictions were varied. In addition, hyperparameter optimization was performed for daily $\text {PM}_{2.5}$ prediction models. In particular, several experiments highlighted the importance of the statistical test for meaningful model comparison.

All in all, we see *evalPM* as a powerful tool for experts in the field of environmental modeling. Given a certain data set or use case, researchers as well as practitioners are able to test prediction models in all their facets. The requirements of the framework are defined in alignment with real-world use cases. Therefore, we argue that it can help consolidate different works and new advancements in the area of air pollutant research.

## Data Availability

The analyzed data sets and produced code from the study are available in the *evalPM* GitHub repository, https://github.com/db-tu-dresden/evalPM and as a PyPI package *evalPM*, https://pypi.org/project/evalPM/.

## Footnotes

1 https://fairmode.jrc.ec.europa.eu/

2 https://scikit-learn.org/stable/

3 https://www.tensorflow.org/

4 https://deep-significance.readthedocs.io/en/latest/

5 https://github.com/db-tu-dresden/evalPM

6 https://pypi.org/project/evalPM/
